# Isolation and characterization of a β-galactosidase from *Lactobacillus helveticus* for industrial processing

**DOI:** 10.3168/jdsc.2024-0563

**Published:** 2024-06-13

**Authors:** Silvette Ruiz-Ramírez, Rafael Jiménez-Flores

**Affiliations:** Department of Food Science and Technology, The Ohio State University, Columbus, OH 43210

## Abstract

In this study, a thermostable β-galactosidase from *Lactobacillus helveticus* OSU-PECh-4A has been isolated through diafiltration and size-exclusion chromatography. The enzyme consists of a heterodimer with a molecular mass of 110 kDa, with a small and large subunit of 36 and 74 kDa, respectively. The Michaelis constant (K_m_) and maximum velocity (V_max_) values for lactose and *o*-nitrophenyl-β-d-galactopyranoside (*o*NPG) hydrolysis were, respectively, 29.87 ± 1.05 m*M*, 1.88 ± 0.02 μmol d-glucose released per min per mg of protein, and 0.067 ± 0.003 m*M*, 1.70 ± 0.05 μmol *o*-nitrophenol (*o*NP) released per min per mg of protein. This β-galactosidase is significantly activated by Mg^+2^ (2–10 m*M*) and slightly inhibited by d-glucose. The enzyme can also hydrolyze 57 ± 3% of lactose after 12 h of reaction at 45°C and under a high concentration of lactose. We propose that this enzyme provides an important advantage from a practical and consumer point of view due to its origins as a probiotic source and improved features for important industrial applications, such as lactose hydrolysis and the potential to produce galacto-oligosaccharides.

## Highlights

• β-Galactosidase has activity at high temperatures (60°C-70°C).
• β-Galactosidase is stable under refrigeration, room, and high temperatures (45°C).
• The enzyme is stable under a range of pH and in the presence of several ions.
• Under optimal conditions, β-Galactosidase hydrolyzes 58% of total lactose.
• The enzyme could be used to produce prebiotics or to valorize dairy byproducts.

## Graphical Abstract

**Figure:**
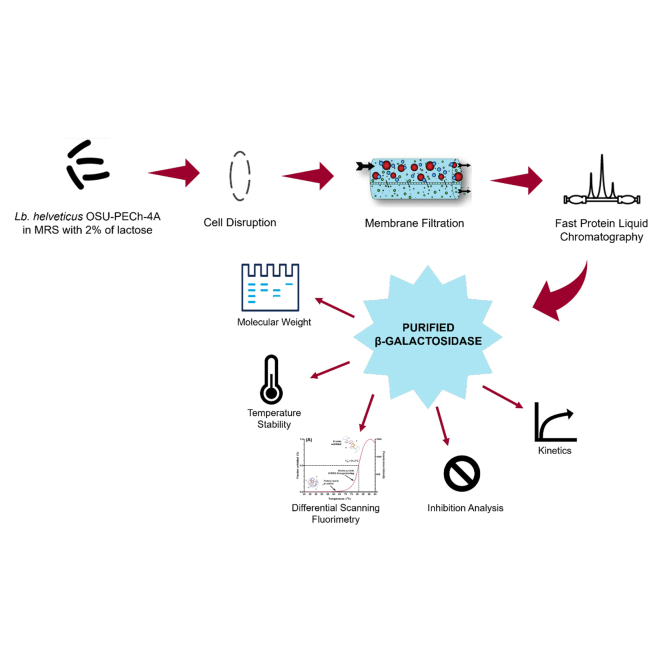
**Summary:** The isolation of β-galactosidase from lactobacilli sources such as *Lactobacillus helveticus* OSU-PECh-4A could provide an opportunity for scientists and the industry looking for β-galactosidase with improved characteristics. Following the growth of *Lb. helveticus* in modified de Man, Rogosa, and Sharpe (MRS) medium containing lactose as the carbon source, the enzyme was purified through a 2-step purification protocol consisting of membrane filtration and size-exclusion chromatography. Once purified, the enzyme was fully characterized through proteomic analysis, steady-state kinetics, and temperature stability, among others. The hydrolysis capacity was evaluated by exposing the β-galactosidase to high lactose concentration under optimal conditions. The enzyme showed a molecular weight and kinetic parameters comparable to other β-galactosidase isolated from lactobacilli sources. However, it has activity at higher temperatures, stability at several temperatures, high hydrolysis capacity, and low end product inhibition. This β-galactosidase could provide several advantages and potential for industrial production, such as lactose hydrolysis and prebiotic production.

*Lactobacillus helveticus* is a homofermentative thermophilic lactic acid bacteria (**LAB**) considered a probiotic and generally recognized as safe (**GRAS**), with the ability to grow at relatively high temperatures. Orla-Jensen first described it in 1919 as an isolate from Emmental cheese (Naser et al., 2006). Its remarkable properties make *Lb. helveticus* one of the most industrially important *Lactobacillus* species. This microorganism can be traditionally found in dairy starter cultures used for the manufacture of fermented dairy products, especially Swiss-type and Italian cheeses such as Gruyere, Grana Padano, and Parmigiano Reggiano, among others (Gatti et al., 2004). *Lactobacillus helveticus* can also be found in other fermented foods, such as wine and sourdough, and in more elaborate environments, such as plants and the human gastrointestinal tract (Giraffa, 2014).

β-Galactosidase isolated from lactobacilli, especially probiotics, has become popular among researchers and the dairy industry for having high activity and high stability, being food-grade, and for the microorganism's easy and exponential growth. β-Galactosidases are enzymes that catalyze the hydrolysis of lactose into its monomers, glucose, and galactose when water is the acceptor molecule (Brás et al., 2010). However, under specific conditions, β-galactosidase can also participate in the transglycosylation of lactose, producing highly demanded prebiotics known as galactose-oligosaccharides (**GOS**; Lu et al., 2020).

Although most of the commercially available β-galactosidase are from sources such as *Aspergillus oryzae*, *Bacillus circulans*, and *Kluyveromyces lactis*, comparisons have been made with lactobacilli-isolated enzymes showing similar properties, increasing its potential utilization in industry with the additional benefit of being GRAS in human food applications (Ruiz-Ramírez and Jiménez-Flores, 2023).

In 2022, we screened and characterized the β-galactosidase specific activity of 15 LAB species. Some of the species screened included *Lactobacillus acidophilus*, *Lacticaseibacillus casei*, *Lactobacillus crispatus*, *Lactobacillus johnsonii*, *Lacticaseibacillus paracasei*, *Lactiplantibacillus pentosus*, and *Lacticaseibacillus rhamnosus*, among others. The study reported that *Lb. helveticus* OSU-PECh-4A possesses remarkably high specific activity compared with the other 24 lactobacilli species from the OSU_PECh collection when cultured in acid whey compared with traditional de Man, Rogosa, and Sharpe (MRS) medium. An increase in the gene expression of β-galactosidase when acid whey was used as the medium, regardless of the specific activity, was also observed, demonstrating the impact of using lactose in the medium (Kolev et al., 2022). The finding also led to the whole genome sequencing of *Lb. helveticus* OSU-PECh-4A, where 4 genes were identified for the production of β-galactosidase, including the overlapping genes LacL and LacM (García-Cano et al., 2022). These *LacLM* genes have been commonly reported in *Lactobacillus* species and are being study due to their transglycosylation potential where moderate lactose conversion has been observed (Ruiz-Ramírez and Jiménez-Flores, 2023).

Therefore, this study aims to evaluate the properties and capacity of β-galactosidase from *Lb. helveticus* to help assess its usefulness in the industry.

For the study, *Lactobacillus helveticus* OSU-PECh-4A (GenBank Accession No. JAHWBM000000000 and MW810614.1) was obtained from the Ohio State University Parker Chair collection (Columbus, OH). The bacterial culture was grown overnight at 37°C in modified de Man, Rogosa, and Sharpe (**mMRS**) broth containing 2% (wt/vol) of lactose as the carbon source to increase β-galactosidase activity.

To isolate the enzyme, *Lb. helveticus OSU*-PECh-4A was cultured in 100 mL of mMRS using overnight culture and incubated at 37°C for 48 h. After incubation, cells were harvested by centrifugation at 3,300 × *g* for 10 min at 4°C, washed twice, and resuspended in 50 mL of 50 m*M* sodium phosphate with 2 m*M* MgCl_2_ (pH 7.0). Cell disruption was conducted with a Branson Sonifier 450 (VWR Scientific Radnor, PA), and cell debris was removed by centrifugation at 3,300 × *g* for 10 min at 4°C. The supernatant, termed “intracellular extract,” was diafiltrated using a 100 kDa cut-off membrane on the Amicon Stirred Cells System (Millipore Corp., Billerica, MA). The retentate was collected, filtered through a 0.22-μm regenerated cellulose filter, and stored at 4°C for size-exclusion chromatography. A sample volume of 1 mL was applied to an Enrich SEC60 column (10 × 300 mm) using the NGC medium-pressure chromatography system (Bio-Rad Laboratories Inc., CA). The column was pre-equilibrated with buffer A (50 m*M* Tris-HCl with 2 m*M* MgCl_2_, 7.0). Proteins were eluted at room temperature at a rate of 0.125 mL/min using a linear gradient of buffer A. Fractions of 500 μL were collected, and the most active was used for further analysis. After each purification step, protein concentration was determined following Bradford's method, using BSA as a standard.

To confirm protein identity and molecular weight, native PAGE and denaturing SDS-PAGE were conducted on a 4% to 10% hand-cast polyacrylamide gel. Coomassie blue staining was used for the visualization of bands. Liquid chromatography-MS coupled with electrospray ionization (ESI-TRAP) identified proteins of interest, where the protein identification was checked manually. Proteins with a Mascot score of 50 or higher with a minimum of 2 unique peptides from one protein with a -b or -y ion sequence tag of 5 residues or better were accepted (proteomic analysis by OSU-Campus Chemical Center).

The β-galactosidase activity was determined using *o*-nitrophenyl-β-d-galactopyranoside (***o*NPG**) and lactose as the substrate, as previously described by Nguyen et al. (2006) with some modifications. When chromogenic *o*NPG was used as the substrate, the enzyme activity was carried out at 37°C using 22 m*M* *o*NPG in 50 m*M* sodium phosphate buffer (pH 7.0) and 100 μL of enzyme sample. After 10 min of incubation, 1 *M* sodium carbonate buffer was added to quench the reaction. The release of *o*-nitrophenol (*o*NP) was assessed by measuring the absorbance at 420 nm. One unit of *o*NPG activity was defined as the amount of micromoles of *o*NP released per minute under the assay conditions described above. When the natural substrate, lactose, was used, 600 m*M* lactose in 50 m*M* sodium phosphate (pH 7.0) was added to the enzyme sample and incubated for 10 min at 37°C. The reaction was stopped by boiling the sample at 99°C for 5 min. The released glucose was measured with an RQflex 20 reflectometer (Millipore Sigma, Darmstadt, Germany). One unit of lactose activity was defined as the amount of micromoles of d-glucose released per minute under the described conditions.

To determine the steady-state kinetic, *o*NPG and lactose were used as substrates, as previously described by Nguyen et al. (2006). The K_cat_ value was calculated on the theoretical maximum velocity (**V_max_**) experimentally determined by nonlinear regression and using a molecular mass of 110 kDa for the catalytically active subunit. Kinetic parameters were then calculated using nonlinear regression and the Henri-Michaelis-Menten equation using GraphPad Prism 10 Software (San Diego, CA). To better understand the enzyme kinetics, the inhibition effect of *o*NPG hydrolysis by d-glucose and the inhibition of lactose hydrolysis by d-galactose were studied following the methodology of Nguyen et al. (2006).

Using a fluorescence-based thermal shift assay, the effect of various pH values and ions on the stability of the protein was studied. Temperature range and fluorescence detection were performed using a CFX96 Touch Real-Time PCR Detection System (Bio-Rad, CA), with fluorescence (GloMelt, Biotium Inc., CA) detected using FAM/SYBR filter set. Thermal denaturation was performed using the following conditions: 30 s at 25°C, followed by a ramp profile from 25 to 99°C at a rate of 0.1°C/s. Each differential scanning fluorimetry (**DSF**) assay contained 10 μL of the enzyme (∼1 µg) and 10 μL of each screen reagent for a final reaction volume of 20 μL. The PEG/pH and PEG/pH 2 kits with 48 different conditions each (Hampton Research, Aliso Viejo, CA) were used to analyze pH conditions. The PEG/Ion Screen kit with 48 different conditions (Hampton Research, Aliso Viejo, CA) consisting of salts, ions, and polymers was used to analyze various additives. The working fluorophore solution (200× in 0.1% DMSO) was added directly to the enzyme for a final concentration of 2×. Each run's control included the enzyme and purification buffer (50 m*M* sodium phosphate, pH 7.0). The protein's thermal stability is reported as the protein's melting temperature (**T_m_**; temperature where the protein is 50% unfolded) by fitting the data in the Boltzmann equation using GraphPad Prism 10 Software.

The β-galactosidase activity temperature dependence and stability were performed by incubating the enzyme in 50 m*M* sodium phosphate buffer (pH 7.0) at various temperatures (25°C–90°C) for 8 h. At specific time intervals, samples were withdrawn, and the residual β-galactosidase specific activity was measured under the standard assay conditions using *o*NPG as the substrate.

To examine whether the high thermostability can be exploited for GOS synthesis, β-galactosidase (containing 0.600 µg/mL of protein with specific activity of 1 µmol per min per mg of protein) was added to 15 mL of lactose solution (205 g/L in 50 m*M* of sodium phosphate [pH 6.6] with 2 m*M* MgCl_2_) and incubated at 45 ± 1°C for 14 h under constant agitation. At 2 h intervals, samples (500 µL) were withdrawn from incubation and heated to 99°C for 5 min in a block heater (VWR, Troemner) to inactivate the enzyme and stop the reaction. The amount of glucose released was measured following the manufacturer's instructions for the glucose oxidase activity kit (Sigma Aldrich, MO).

All results are expressed as the mean ± SD of at least 3 replicates. Statistical differences between pH and ions T_m_ were analyzed by one-way ANOVA followed by Tukey as a post hoc test to mean comparisons considering α = 0.05 using JMP 16 Software (SAS Institute Inc., NC).

β-Galactosidase was concentrated to apparent homogeneity to an 800-fold protein purity as compared with the extract by decreasing the protein concentration from 36 mg to 56 µg, obtaining a recovery yield of 72% and a final specific activity of 44.6 U/mg of protein. Unlike other methods used for the isolation of β-galactosidase such as ammonium sulfate precipitation, the protocol used for this enzyme eliminates the use of salt and chemicals that could interact with the protein structure, thus preserving the enzymatic activity while reducing purification cost and time. This also minimized the generation of any chemical waste, providing the industry with a more sustainable method.

The proteomic analysis identified 13 peptide sequences belonging to a heterodimer from the GH2 family, consisting of a large subunit and small subunit of 74 kDa (gene location: JAHWBM010000062.1:187–2073; CDS: MBW1220442.1) and 36 kDa (gene location: JAHWBM010000062.1:2057–3013; CDS: MBW1220443.1). Based on native PAGE, the enzyme's molecular mass is approximately 110 kDa compared with reference proteins, respectively. This results demonstrates that out of the 4 β-galactosidase genes found on *Lb. helveticus* OSU-PECh-4A genome (García-Cano et al., 2022), the isolated enzyme belongs to the *LacLM* family. These subunits are remarkably similar to those reported by other heterodimeric β-galactosidases of *LacLM* type from the *Lactobacillaceae* family, such as *Limosilactobacillus reuteri* (Nguyen et al., 2006), *Lactiplantibacillus pentosus* (Maischberger et al., 2010), *Lactobacillus acidophilus* (Nguyen et al., 2007), *Limosilactobacillus fermentum* (Liu et al., 2011), and even on another strain of *Lactobacillus helveticus* (Kittibunchakul et al., 2019).

The calculated Michaelis constant (**K_m_**), V_max_, and K_cat_ for the hydrolysis of lactose and *o*NPG are 29.87 ± 1.05 m*M*, 1.88 ± 0.02 μmol min^−1^ mg^−1^, 138.7 ± 1.6 s^−1^ and 67 ± 0.003 μ*M*, 1.7 ± 0.05 μmol min^−1^ mg^−1^, 103.5 s^−1^, respectively. A similar K_m_ for lactose has been reported for β-galactosidase from *Lactiplantibacillus plantarum* (29 m*M*), *Lim. reuteri* (31 m*M*), and *Lim. fermentum* (27 m*M*; Ruiz-Ramírez and Jiménez-Flores, 2023). The K_cat_ shows that the enzyme is highly efficient when using the natural substrate, lactose, because it produces more products per enzyme per second. The Henri-Michaelis-Menten plots also showed that β-galactosidase is inhibited by low concentration (≥3 m*M*) of *o*NPG. A similar effect was reported on the β-galactosidase from *Lactobacillus sakei*, which was inhibited by 5 m*M* *o*NPG (Iqbal et al., 2011), as well as by the reaction end products, d-glucose and d-galactose.

In this study, β-galactosidase loses 30% of the initial activity when the end product, d-galactose, is present in concentrations as low as 1 m*M*, demonstrating a slight inhibition. However, the enzyme retains the other 70% of its activity at higher galactose concentrations (2–200 m*M*), demonstrating some degree of resistance. For the second end product, d-glucose, surprisingly, an allosteric effect was observed in which β-galactosidase activity first decreased by 30% when concentration was lower than 50 m*M* and then increased by 20% at higher concentration (200 m*M*), losing a total of 10% of activity when compared with the initial activity. This finding is consistent with allosteric positive cooperativity, which, to the best of our knowledge, has not been reported before. The allosteric effect and resistance to high glucose concentrations could overcome one of the most reported limitations of lactobacilli β-galactosidase. It can also be a great advantage during the manufacture of lactose-free products or for high yield of prebiotics due to inhibition of end products continuing to be a severe limitation of these enzymes' activity.

The optimal temperature in terms of β-galactosidase activity from *Lb. helveticus* for the hydrolysis of lactose and *o*NPG under the 10 min assay is 60°C–70°C and 70°C–80°C, respectively, demonstrating thermophilic properties (Figure 1A). Thermophilic enzymes are highly desired for industrial applications, especially β-galactosidase because the formation of GOS is favorable over hydrolysis at high temperatures (Gosling et al., 2010). High temperatures are also highly important due to high reaction velocity, low risk of contamination, and low product inhibition. The enzyme is stable for 30 min at 60°C to 70°C before 80% of its initial activity is lost and at 50°C for 4 h before 85% of the activity is lost. Interestingly, β-galactosidase did not significantly change when incubated at 4°C, 40°C, and 45°C (Figure 1B). Although the optimal temperature (∼60°C) is similar to β-galactosidase from *Lpb. plantarum*, *Lactiplantibacillus pentosus*, and *Lactobacillus delbrueckii* ssp. *bulgaricus*, the length of time the enzyme stays stable before inactivation differs greatly. Something similar was observed with *Lpb. plantarum* WCFS1, whose optimal temperature is 55°C to 60°C but is only stable at 37°C for 5 h (Iqbal et al., 2010). This change could be explained by the popular assumption that the dependence of enzyme activity on temperature can be explained only by the effect of temperature on the catalytic reaction and denaturation without considering the equilibrium between the active and inactive form of the enzyme (Daniel et al., 2008). However, based on the thermostability of the *LacLM* β-galactosidase isolated from *Lb. helveticus* OSU-PECh-4A, we hypothesized that the enzyme has potential for lactose hydrolysis at industrial temperatures and for GOS production.

**Figure 1:**
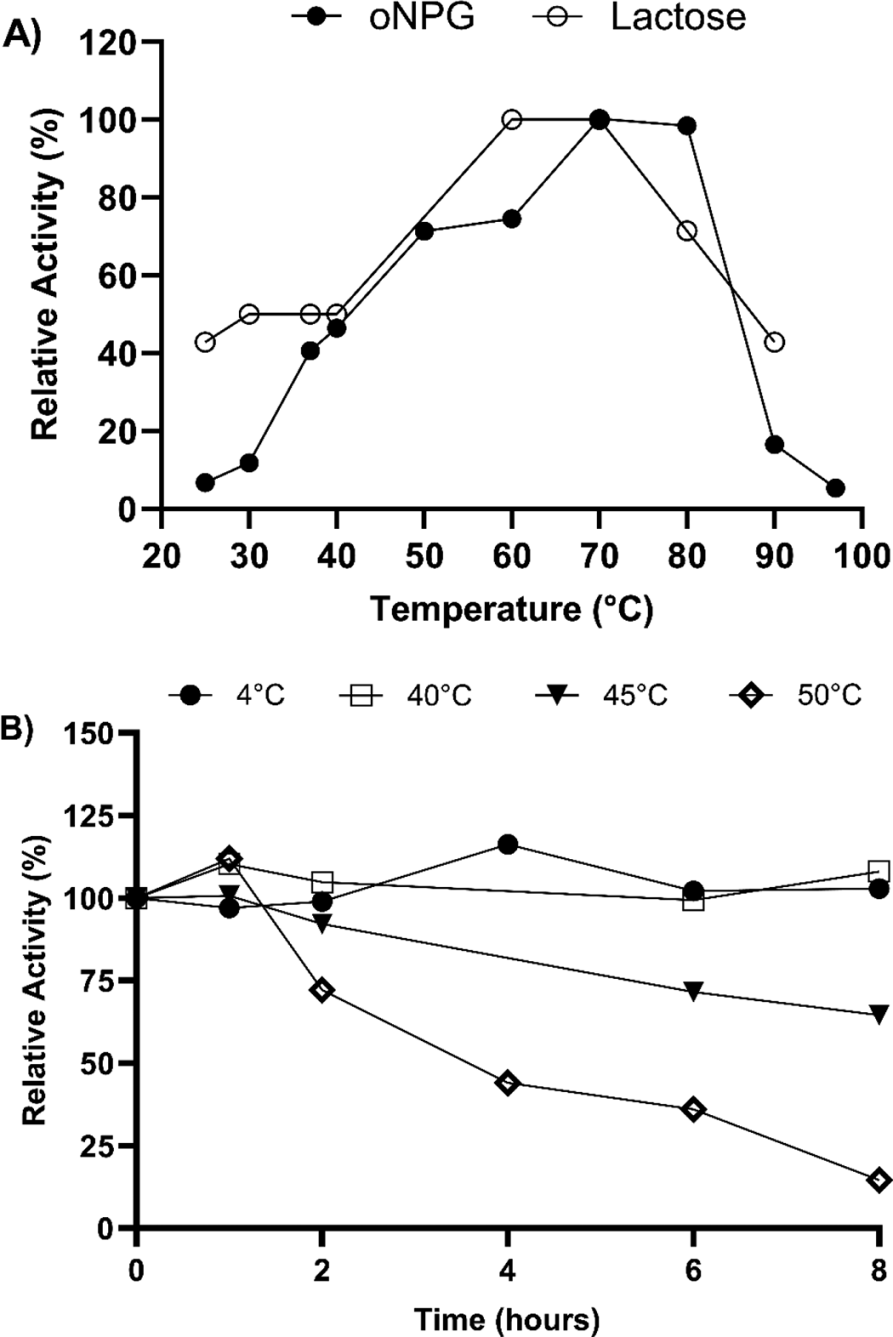
Effect of temperature on the (A) enzyme activity after 10 min and (B) enzyme stability throughout 8 h of incubation at different temperatures using *o*NPG and lactose as substrates. Relative activities are given in comparison to the maximum activities measured under optimal conditions (100%). All data points represent the average value from 3 replicates.

The purified β-galactosidase was further characterized by the effect of pH and different additives on the protein's thermal stability rather than its activity to provide a better insight into the enzyme's structural stability and to identify possible components that could affect or enhance its function. The DSF assays determined that β-galactosidase at pH 7.0 has a T_m_ value of 95.8 ± 0.1°C. Acidic and basic pH did not cause significant structural change (Figure 2A and 2B). However, at pH 7.1, the enzyme has a significantly positive shift when compared with pH 4.4 (*P* < 0.03) and pH 9.2 (*P* < 0.004), where the T_m_ increased from 93.3 ± 0.82°C to 97.1 ± 0.5°C. Interestingly, the β-galactosidase demonstrates a highly stable structural order under a wide range of pH compared with other β-galactosidase from lactobacilli sources, which have been reported to have a narrow range of optimal pH of 6–7 or 6–8 (Ruiz-Ramírez and Jiménez-Flores, 2023). A slight but insignificant increase was observed in T_m_ at a pH of 6.6 compared with control (pH 7.0), suggesting this small range could be more optimal for the enzyme. Some pH conditions did not generate a sigmoidal curve, which we hypothesize could be due to a T_m_ value near or higher than the instrument's limit (99°C).

**Figure 2:**
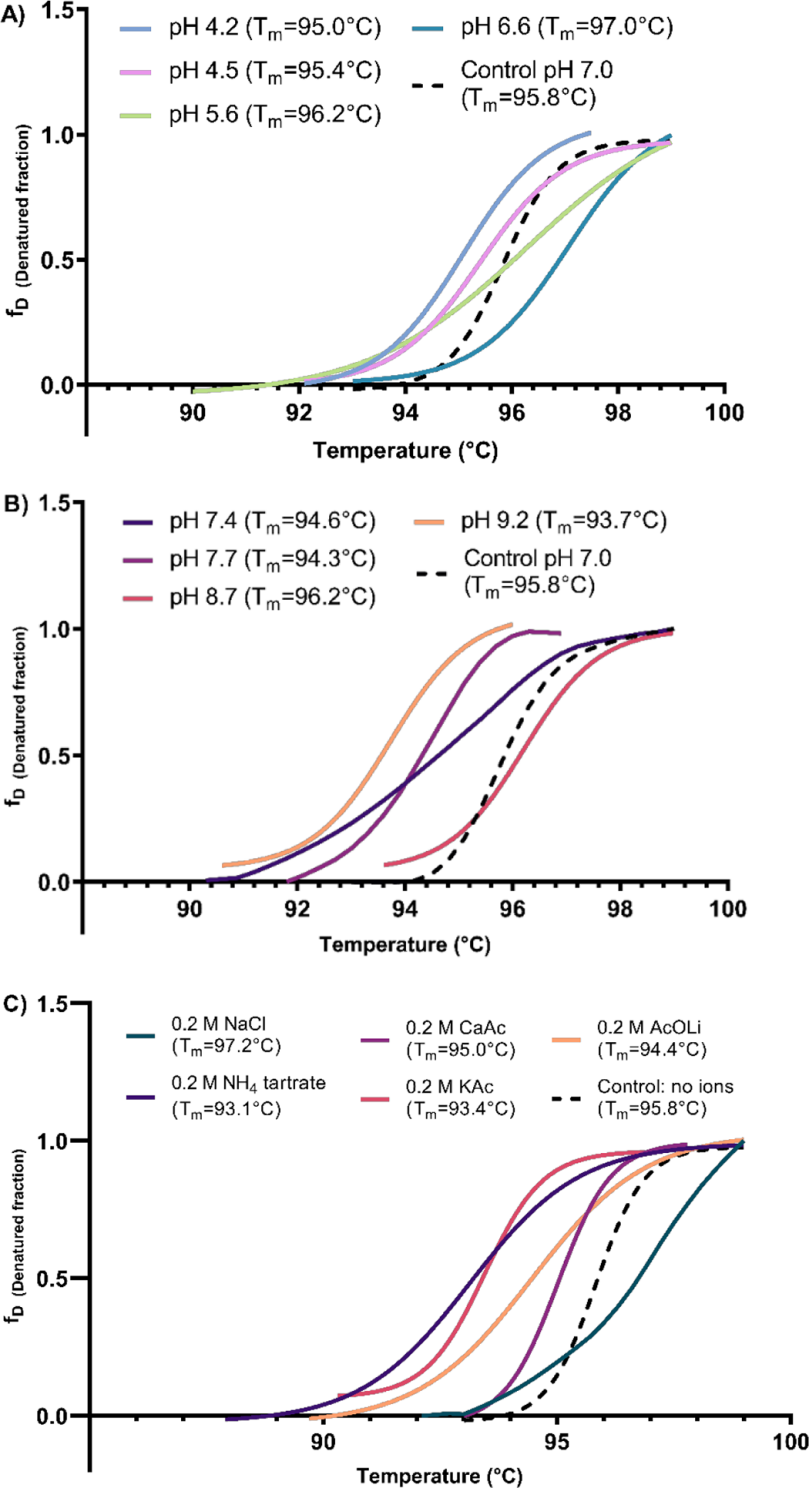
Effect of (A) acidic pH, (B) basic pH, and (C) ions on the β-galactosidase thermal stability of *Lactobacillus helveticus*. All sigmoidal curves represent the average value from 3 individual experiments. The different melting point values (T_m_) were calculated at the inflection point of the transition temperatures using Boltzmann's sigmoidal fit.

In terms of ions and reagents, β-galactosidase without the presence of any ions has a T_m_ value of 98.2 ± 0.12°C, which is not significantly affected by the presence of NaCl (T_m_: 97.4 ± 0.72°C), CaCl (T_m_: 98.1 ± 0.26°C), or ZnAc (T_m_: 97.4 ± 0.72°C). However, the enzyme structure significantly changes (*P* < 0.02) into a more disordered conformation by decreasing to a T_m_ value of 93 ± 1°C in the presence of ammonium tartrate, potassium acetate, ammonium citrate, potassium iodide, and lithium acetate (Figure 2C). Monovalent ions such as sodium have been reported to activate β-galactosidase from lactobacilli sources such as *Levilactobacillus brevis* (Bhalla et al., 2015), *Lim. fermentum* (Liu et al., 2011), and *Lim. reuteri* (Nguyen et al., 2006), whereas divalent ions such as Ca^2+^ and Zn^2+^ are usually reported to reduce β-galactosidase activity (Nguyen et al., 2007, 2012; Kittibunchakul et al., 2019). The structural resistance demonstrated by this strain of *Lb. helveticus* could be explained by the nature of the species, a microorganism used to manufacture cheeses, where Na^+^ and Ca^2+^ are widely present (Slattery et al., 2010). Mg^2+^ significantly increases the enzyme activity (*P* < 0.0001) by 17 ± 1% when concentrations from 2 to 10 m*M* of MgCl_2_ are added to the buffer. Mg^2+^ is commonly reported to activate β-galactosidase 5- to 500-fold (Brás et al., 2010). However, there is no difference between adding 2 to 10 m*M* MgCl_2_. Magnesium has also been reported to promote the hydrolysis of lactose by an electrophilic attack and the stabilization of end products during the galactosylation step (Brás et al., 2010; Zhou et al., 2017), making it an essential cofactor for this enzyme.

Evaluation of the enzyme thermostability through lactose hydrolysis is shown in Figure 3. β-Galactosidase at high lactose concentration (20% wt/vol) can hydrolyze 49 ± 2% of lactose in the first 4 h of reaction and gradually continues until it reaches a maximum of 57 ± 3% at 12 h, demonstrating the enzyme's thermostability. However, after 12 h, the beginning of a plateau can be observed, which could potentially indicate the predomination of transglycosylation over hydrolysis, and therefore, lactose conversion should be further studied.

**Figure 3:**
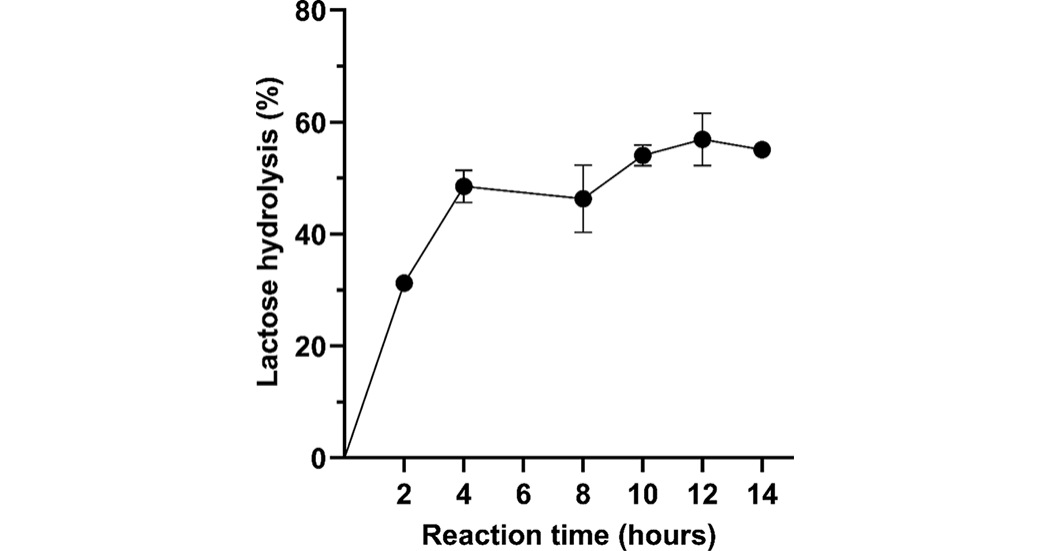
Lactose hydrolysis of β-galactosidase from *Lactobacillus helveticus* OSU-PECh-4A after 14 h reaction at 45°C with an initial lactose concentration of 200 g/L. All data points represent the average value from 3 individual experiments. Line bars represent SD of at least 3 replicates.

In conclusion, we have successfully isolated and characterized a β-galactosidase from the potential probiotic *Lb. helveticus* OSU-PECh-4A using a 2-step, low-cost, and less time-consuming purification process for industry settings. The enzyme showed stability at high temperatures, a wide pH range, and low end product inhibition. It also presents the potential of β-galactosidase with high hydrolysis capacity and the advantage of introducing probiotic enzymes in industrial processes. Using the enzyme optimal characteristics, future work includes the evaluation of GOS production as well as the opportunity to use the microorganism as a source of a highly functional β-galactosidase in dairy products.
